# Different expression patterns of inflammatory cytokines induced by lipopolysaccharides from *Escherichia coli* or *Porphyromonas gingivalis* in human dental pulp stem cells

**DOI:** 10.1186/s12903-022-02161-x

**Published:** 2022-04-12

**Authors:** Chunhua Lan, Shuai Chen, Shan Jiang, Huaxiang Lei, Zhiyu Cai, Xiaojing Huang

**Affiliations:** 1 Fujian Key Laboratory of Oral Diseases & Fujian Provincial Engineering Research Center of Oral Biomaterial & Stomatological Key Lab of Fujian College and University, School and Hospital of Stomatology, Fujian Medical University, 246 Yangqiao Zhong Road, Fuzhou, 350002 China; 2 Institute of Stomatology & Research Center of Dental and Craniofacial Implants, School and Hospital of Stomatology, Fujian Medical University, Fuzhou, China; 3 Southern Medical University, Shenzhen Stomatology Hospital (Pingshan), Shenzhen, China; 4 Department of Stomatology, Fujian Medical University Union Hospital, Fuzhou, China

**Keywords:** Lipopolysaccharide, Pulpitis, Inflammation mediators, Human dental pulp stem cells, *Escherichia coli*, *Porphyromonas gingivalis*

## Abstract

**Background:**

Lipopolysaccharide (LPS) is one of the leading causes of pulpitis. The differences in establishing an in vitro pulpitis model by using different lipopolysaccharides (LPSs) are unknown. This study aimed to determine the discrepancy in the ability to induce the expression of inflammatory cytokines and the underlying mechanism between *Escherichia coli* (*E. coli*) and *Porphyromonas gingivalis* (*P. gingivalis*) LPSs in human dental pulp stem cells (hDPSCs).

**Material and methods:**

Quantitative real-time polymerase chain reaction (QRT-PCR) was used to evaluate the mRNA levels of inflammatory cytokines including IL-6, IL-8, COX-2, IL-1β, and TNF-α expressed by hDPSCs at each time point. ELISA was used to assess the interleukin-6 (IL-6) protein level. The role of toll-like receptors (TLR)2 and TLR4 in the inflammatory response in hDPSCs initiated by LPSs was assessed by QRT-PCR and flow cytometry.

**Results:**

The *E. coli* LPS significantly enhanced the mRNA expression of inflammatory cytokines and the production of the IL-6 protein (*p* < 0.05) in hDPSCs. The peaks of all observed inflammation mediators’ expression in hDPSCs were reached 3–12 h after stimulation by 1 μg/mL *E. coli* LPS. *E. coli* LPS enhanced the TLR4 expression (*p* < 0.05) but not TLR2 in hDPSCs, whereas *P. gingivalis* LPS did not affect TLR2 or TLR4 expression in hDPSCs. The TLR4 inhibitor pretreatment significantly inhibited the gene expression of inflammatory cytokines upregulated by *E. coli* LPS (*p* < 0.05).

**Conclusion:**

Under the condition of this study, *E. coli* LPS but not *P. gingivalis* LPS is effective in promoting the expression of inflammatory cytokines by hDPSCs. *E. coli* LPS increases the TLR4 expression in hDPSCs. *P. gingivalis* LPS has no effect on TLR2 or TLR4 expression in hDPSCs.

**Supplementary Information:**

The online version contains supplementary material available at 10.1186/s12903-022-02161-x.

## Introduction

Pulpitis is an inflammatory pathosis of pulp tissue in response to various external stimuli primarily caused by bacterial infection. As a richly vascularized and innervated connective tissue, dental pulp is composed of diverse cell populations, among which dental pulp stem cells (DPSCs) are pivotal for their highly proliferative potential, self-renewal capability, and multilineage differentiation aptitude [1], DPSCs continuously replenish odontoblasts to form secondary and tertiary dentin throughout adult life and in reaction to insults [2]. Upon stimulation of lipopolysaccharides (LPSs), DPSCs could be recruited from their niche, migrate to the site of inflammation, and differentiate into odontoblast-like cells to form reparative dentin. LPS has also been reported to be involved in mesenchymal stem cells (MSCs) differentiation and inflammatory responses [3]. It has been reported that human dental pulp stem cells (hDPSCs) from carious teeth manifested enhanced proliferation and osteogenic differentiation in comparison with their counterparts from healthy teeth [4]. DPSCs is regarded as a readily available source of multipotent stromal cells for tissue regeneration. DPSCs also involved in modulation of pulp inflammation [5]. Recent evidences have revealed that DPSCs could modulate the secretion of inflammatory cytokines and participate in the host immune response [6–8]. The immunomodulatory potential of DPSCs may be of particular importance for pulp tissue to repair or regenerate under conditions of pulpitis. To date, many studies have focused on the role of DPSCs in the progression and treatment of pulpitis via establishing in vitro pulpitis models to simulate an inflammatory environment of DPSCs [7, 9, 10].

Toll-like receptors (TLRs) are pattern recognition receptors sensing specific pathogen-associated molecular patterns (PAMPs), connecting innate and adaptive immunity. TLRs are crucial in pathogenesis of chronic inflammatory, autoimmune, and infectious diseases [11]. So far, 10 functional human TLRs have been identified. Among them, TLR2 and TLR4 are extracellular TLRs which could recognize peptidoglycans and lipoteichoic acid (LTA) of Gram-positive bacteria [12, 13] and lipopolysaccharide (LPS) primarily from Gram-negative bacteria [14] respectively. When cultured in vitro, DPSCs express TLRs 1–10 at differential levels, with TLR2 and TLR4 in significant amounts, making them susceptible to LPS or LTA [15].

LPS, composed of lipids and polysaccharides, is a major component of the membrane of gram-negative bacteria that causes cell inflammation [16, 17]. By binding to TLRs of the cell, LPS activates various downstream signaling pathways, leading to the synthesis of inflammation mediators, such as interleukin (IL)-1β, tumor necrosis factor-alpha (TNF-α), IL-6, IL-8, and cyclooxygenase-2 (COX-2) [18, 19]. Being the critical initiator in pulpitis pathogenesis, bacterial LPS penetrates into the affected dental pulp tissue, motivates substantial release of inflammatory mediators from dental pulp, such as IL-1β, TNF-α, IL-6, and IL-8 [20, 21], thus triggering the inflammatory response of the dental pulp [22, 23]. The inflammation-inducing effects of LPS varies among different bacterial sources and different target cells. Nebel et al. compared the IL-6 gene and protein production of human periodontal ligament cells (hPDLCs) upon stimulation by LPSs from *Escherichia coli* (*E. coli)* and *Porphyromonas gingivalis* (*P. gingivalis*) [24]. They found that *E. coli* LPS enhances the IL-6 expression dramatically, whereas *P. gingivalis* LPS has no effect on hPDLCs. In another study, gingival fibroblast cells are reported to be more sensitive to *E. coli* LPS than to *P. gingivalis* LPS in the expression of inducible nitric oxide, IL-6, and monocyte chemotactic protein-1 (MCP-1) [25]. By contrast, macrophages manifest a more robust inflammatory reaction in expression of of IL-1β, IL-6, and MCP-1 in response to *P. gingivalis* LPS in comparison with *E. coli* LPS [25]. In a study conducted by Palaska et al., no significant difference in inflammatory response of human mast cells between *P. gingivalis* LPS and *E. coli* LPS was observed [26]. Obviously, the inflammation-inducing impact of LPS on target cells is both bacteria-specific and cell-specific.

Different stimuli such as LPS, TNF, bacterial extracts are used to imitate an inflammatory dental pulp microenvironment [27–29]. To stimulate DPSCs in establishing in vitro pulpitis models, many researchers use *E. coli* LPS [10, 30] whereas others use *P. gingivalis* LPS [27, 31]. LPS from *E. coli*, targets TLR4 and activates the downstream NF-κB signaling pathway, leading to the expression of inflammatory cytokines [32]. The interaction of *P. gingivalis* LPS with TLR2 or TLR4 remains controversial [33]. The TLR2 activity of *P. gingivalis* LPS might be caused by a contaminant lipoprotein [34]. As LPSs of different bacteria have been used in these studies, it is imperative to understand the discrepancy of the inflammation-inducing property between *E. coli* and *P. gingivalis* LPSs when interpretting and comparing these results. According to a most recently published systematic review [35], despite 105 in vitro studies using LPS in induction of pulp cell inflammation have been reported so far, only 2 experiments adopted both *E. coli* and *P. gingivalis* LPSs in stimulating heterogenous dental pulp cells [36, 37]. Moreover, scarce evidence exists comparing the inflammatory effects of *E. coli* and *P. gingivalis* LPS on DPSCs. Thus, our study aims to determine the differences in the ability to induce the expression of inflammatory cytokines over time by hDPSCs between *E. coli* and *P. gingivalis* LPSs. Furthermore, we have investigated the role of TLR4 and TLR2 in hDPSCs response to *E. coli* and *P. gingivalis* LPS-induced inflammation. The hypothesis is that the LPS from *E. coli* is more potent than the LPS from *P. gingivalis* in eliciting inflammatory reactions in hDPSCs. The LPSs could induce proinflammatory expression in hDPSCs via TLR4. The novelty of this study is to provide comparative data of the inflammation-inducing capacitity between *E. coli* and *P. gingivalis* LPSs on hDPSCs.

## Materials & methods

### Cell isolation and culture

We collected impacted molars without caries from healthy volunteers aged 18 to 25 years. The procedures of collecting the extracted teeth were under the Committee of Ethics of School and Hospital of Stomatology, Fujian Medical University (No.201652), and informed consent was obtained. Immediately after extraction, each tooth was fractured into several parts by pliers (bone forceps) under sterile conditions. The dental pulp tissue from the teeth was isolated and collected into the Eppendorf tube. As described in the previous study, the pulp tissue was minced into 1 × 1 mm^2^ fragments and digested with a mix of type I collagenase (3 mg/ml) and dispase (4 mg/ml; Sigma -Aldrich, St Louis, MO, USA) for 30–60 min at 37 °C [38]. Next, we obtained a single-cell suspension using a 70 mm cell strainer to filter solutions[38]. The suspension was then transferred onto a 6 cm culture dish, and cultured in an incubator maintained at 37 °C containing 5% carbon dioxide. The minimum essential medium with 10% fetal bovine serum (FBS, Gibco BRL, Rockville, MD, USA), 100 mg/ml streptomycin (Gibco BRL), and 100 units/ml penicillin (Gibco BRL) was used as culture medium. We used the third and fourth passage cells in subsequent experiments.

### Characterization of hDPSCs

Following the method in a previous study [39], the mesenchymal antigen markers of the cells were identified using flow cytometry. The fluorescently conjugated antibodies used were as follows (eBioscience, San Diego, CA, USA): anti-CD90-allophycocyanin (APC), anti-CD105-phycoerythrin (PE), anti-CD73-PE, anti-CD146-PE, anti-CD45-APC, and anti-CD34-fluorescein isothiocyanate (FITC). Correspondingly conjugated isotype control included mouse IgG-APC, IgG-PE, and IgG-FITC.

The cells were cultured in an osteogenic induction medium supplemented with 10 nM dexamethasone, 0.2 mM ascorbic acid-2-phosphate, and 10 mM sodium β-glycerophosphate (Sigma-Aldrich) for osteogenic differentiation for 3 weeks. Then the culture was fixed with 4% paraformaldehyde for 30 min and stained with 2% Alizarin Red.

The cells were cultured in the adipogenic induction medium (Cyagen, Santa Clara, CA, USA) for 3 weeks and then stained with oil red “O” solution (Sigma-Aldrich) to test the adipogenic differentiation ability.

The colony-forming unit (CFU) test was carried out to determine the self-renewal potential of the isolated cells. Briefly, 1000 cells per well were seeded in a 6-well dish and cultured in the growth medium. The culture medium was changed every 3 days. After 14 days, cells were stained with 0.5% crystal violet solution for 30 min, observed, and photographed using a microscope.

The immunofluorescence staining for specific proteins was performed to detect the origin of cells. The immunofluorescence staining protocol was in accordance with a previous study [40]. Rabbit antihuman vimentin (Abclonal, Woburn, MA, USA) and rabbit antihuman cytokeratin (Abclonal) proteins referred to mesenchymal and epithelial origins. FITC-labeled goat antirabbit IgG (Abcam, Cambridge, UK) was used as the secondary antibody, and DAPI (Solarbio, Beijing, China) was used as nuclear-staining fluorescence.

### LPS treatment and grouping

Upon reaching 80%–90% confluence, hDPSCs were stimulated with LPS of *P. gingivalis* (InvivoGen, Carlsbad, CA, USA)(standard version, # tlrl-pglps) or *E.coli* (Sigma Aldrich) (serotype 055:B5, L5418) at the concentration of 1 μg/mL [41, 42] referring to a previous study [43]. The cells not treated by *E. coli* LPS or *P. gingivalis* LPS were used as the control group. The treated cells at different time points (1.5, 3, 6, 12, and 24 h) were harvested for assessing the mRNA expression of IL-6, IL-8, COX-2, IL-1β, and TNF-α. Besides, we measured the gene levels of TLR4 and TLR2 stimulated by *E. coli* and *P. gingivalis* LPSs (1 μg/mL) at 1.5, 3, 6, 12, and 24 h to investigate the underlying mechanism of LPS-induced inflammation of hDPSCs. Also, we explored the effects of *E. coli* and *P. gingivalis* LPSs (1 μg/mL) on the protein production of TLR4 and TLR2 in hDPSCs by flow cytometry. Furthermore, cells were pretreated with or without 10 μmol/L TAK-242 (HY-11109, MedChem Express, NJ, USA) for 30 min and added with *E. coli* LPS (1 μg/mL) for another 3 h to confirm how TLR4 acted in the inflammatory mediator expression of hDPSCs induced by *E. coli* LPS. Afterward, we collected all cells and evaluated the fluctuation in the gene expression levels of IL-6, IL-8, COX-2, IL-1β, TNF-α, and TLR4.

### Quantitative real-time polymerase chain reaction (QRT-PCR)

Briefly, we extracted the total RNA of hDPSCs by using Trizol (Invitrogen). According to the manufacturer’s protocol, we synthesized the cDNA from 1 μg total RNA using the PrimeScript RT reagent kit with the gDNA Eraser (Takara, Kusatsu, Japan). The primer sequences used in our research are shown in Additional file 1: Table S1. Each cDNA sample was amplified in triplicate on the LightCycler 480 II real-time PCR system using a two-step method. The expression of targeted genes was analyzed by calculating the amount of target cDNA relative to the housekeeping gene glyceraldehyde-3-phosphate dehydrogenase following the 2^−ΔΔCT^ principle.

### Enzyme-linked Immunosorbent Assay (ELISA)

The IL-6 protein released into the cell culture supernatant was measured to assess the influence of *E. coli* and *P. gingivalis* LPSs on the inflammation-inducing ability. The hDPSCs were cultured in triplicate at a density of 5 × 10^4^ per well in 24-well plates in the completed culture medium containing 10% FBS. After reaching approximately 80% confluence, the medium was removed and replaced with a new medium free of serum for 18 h. Then, hDPSCs were stimulated by 1 μg/mL *E. coli* LPS or 1 μg/mL *P. gingivalis* LPS in the completed medium for another 24 h. Supernatants were gathered and stored at 80 °C until further use. By the manufacturer's protocol, we analyzed the IL-6 protein level from culture supernatants using a commercially available human-specific ELISA kit (Neobioscience, Shenzhen, China).

### Flow cytometry

The BD Accuri C6 Software was used to investigate TLR4 and TLR2 expression on the surface of hDPSCs stimulated by LPS (1 μg/mL) from *E. coli* or *P. gingivalis*. Cells were collected, washed with PBS, counted, and then resuspended in the staining buffer. Cells were incubated with the anti-TLR4 antibody (Abcam) (ab13556) or anti-TLR2 antibody (Abcam) (ab213676) for 1 h at 4 °C. The secondary antibody diluted to 1/2000 was added for another 30 min in the dark (Abcam) (ab150079). The isotype control antibody (Abcam) (ab37415) was used under the same conditions. Data analysis was performed using the FlowJo 10.6.2 software.

### Statistical analysis

Data were expressed as a mean ± standard deviation and analyzed using one-way Analysis of variance, followed by Tukey’s test (equal variance) or Dunnett’s T3 (unequal variance). Statistical significance was determined at *p* < 0.05.

## Results

### Characterization results of hDPSCs

**Fig. 1:**
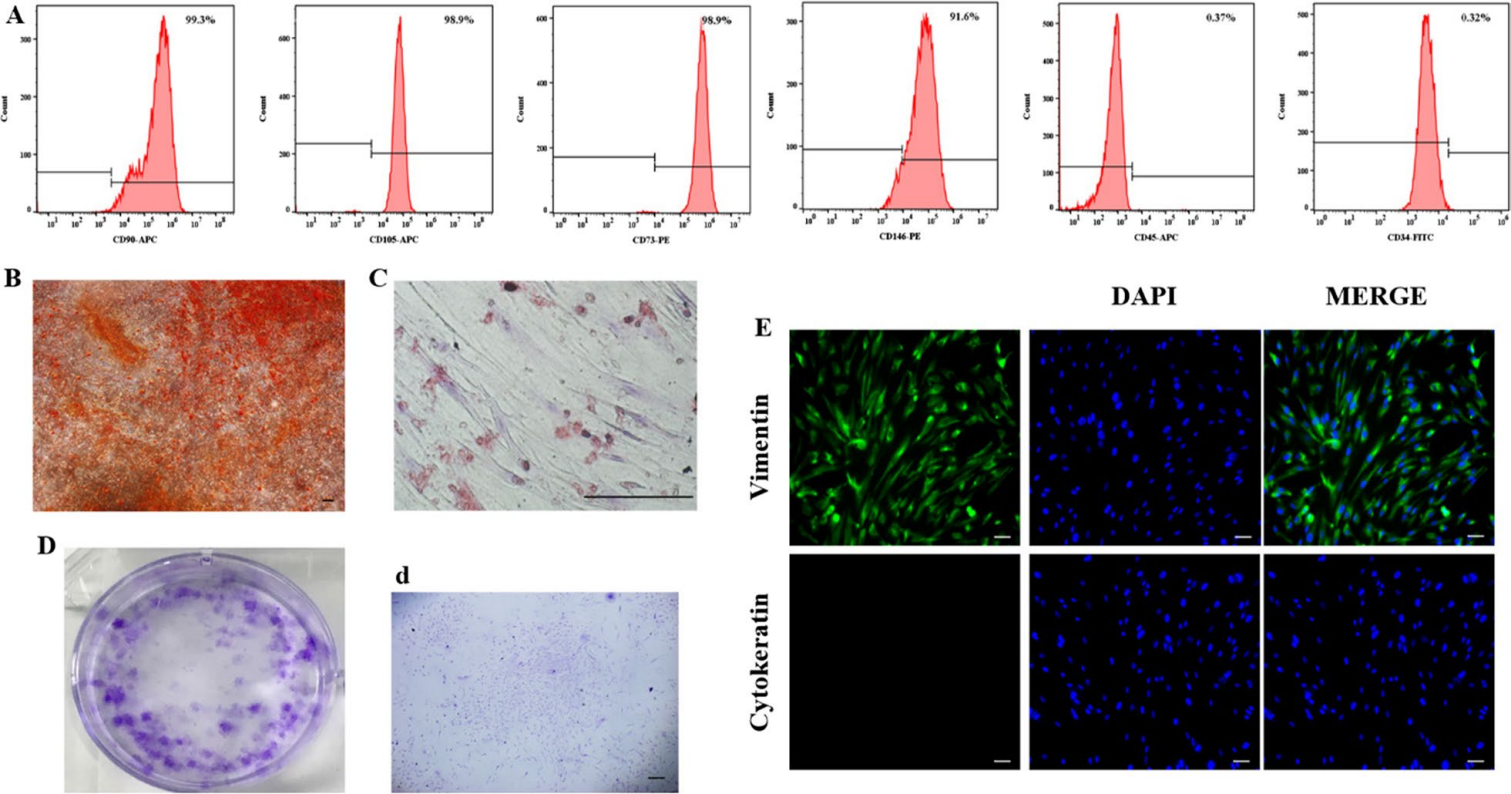
Characterization of hDPSCs. **A** Representative histograms about surface markers on hDPSCs by flow cytometry. **B** Mineralized nodules formed in hDPSCs after osteogenic differentiation for 3 weeks. **C** Lipid droplets after adipogenic induction in hDPSCs for 3 weeks. **D**, **d** Colonies of hDPSCs visualized using crystal violet staining. **E** Positive immunofluorescence to vimentin and negative immunofluorescence to cytokeratin of hDPSCs. All scale bars are equal to 50 μm in **B**–**E**

The flow cytometry showed mesenchymal markers (CD73, CD105, CD90, CD146) positive and hematopoietic markers (CD34, CD45) negative on hDPSCs (Fig. 1A). Many mineralized nodules and several red lipid droplets formed in hDPSCs, respectively (Fig. 1B, C). The CFU test showed prominent colonies in hDPSCs, displaying the apparent self-proliferation capacity of hDPSCs (Fig. 1D, d). The isolated cells were positive for anti-vimentin and negative for anti-cytokeratin, proving that hDPSCs in our study were derived from human mesenchymal cells (Fig. 1E).

### Inflammatory cytokine mRNA expression

**Fig. 2:**
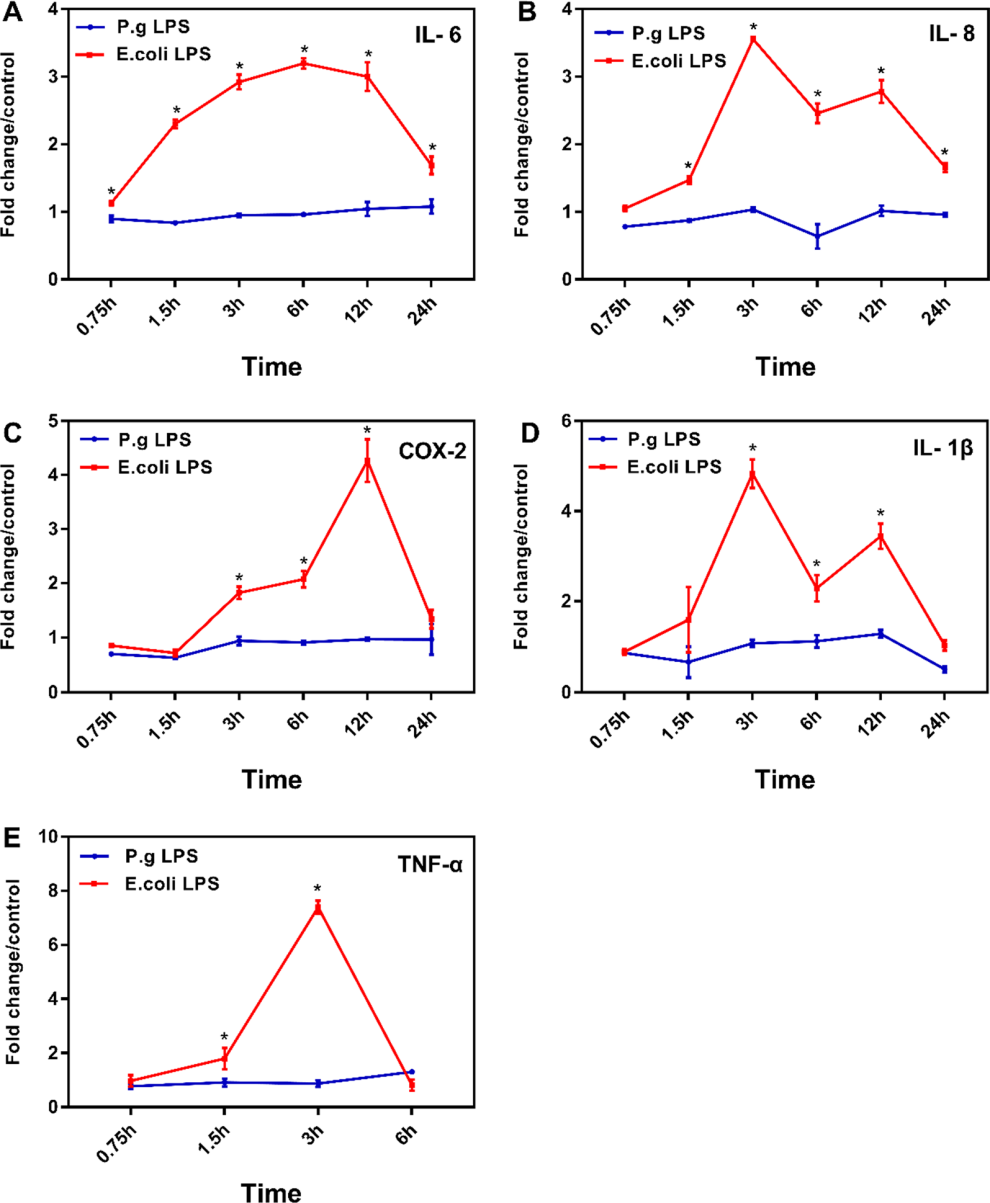
Inflammatory cytokines mRNA expression patterns in hDPSCs triggered by *E. coli* or *P. gingivalis* LPS. **A** IL-6, **B** IL-8, **C** COX-2 **D** IL-1β, and **E** TNF-α mRNA. Cells are stimulated by 1 μg/mL LPS from *E. coli* or *P. gingivalis* for 0.75,1.5, 3, 6, 12, and 24 h, respectively. The cells without LPS treatment (untreated cells) were used as the control group. This figure is a typical one of three independent experiments with three replications for each experiment. Data are shown as mean ± SD (n = 3). Y-axis represents the relative fold expression of inflammatory mediators relative to the control group. *p < 0.05, vs the control group at the same time point

Compared with the untreated cell, hDPSCs stimulated by *E. coli* LPS (1 μg/mL) significantly upregulated IL-6 mRNA expression at all observed time points (*p* < 0.05, Fig. 2A). The IL-8 mRNA level was increased significantly in hDPSCs stimulated by *E. coli* LPS from 1.5 h to 24 h (*p* < 0.05, Fig. 2B). The gene expression levels of COX-2 and IL-1β by hDPSCs were significantly increased from 3 to 12 h in the group stimulated by *E. coli* LPS (*p* < 0.05, Fig. 2C, D). *E. coli* LPS elicited a significant upregulation of TNF-α mRNA in hDPSCs at 1.5 and 3 h (*p* < 0.05, Fig. 2E). However, we detected no IL-6, IL-8, COX-2, IL-1β and TNF-α expression level discrepancy in hDPSCs between the *P. gingivalis* LPS and the control groups at each time point (*p* > 0.05, Fig. 2A–E). In our study, a high concentration of *P. gingivalis* LPS (10 μg/mL) showed no elevated mRNA expression level of proinflammatory cytokines in hDPSCs (Additional file 2: Fig. S1).

In general, only the LPS from *E. coli* notably improved IL-8, IL-6, COX-2, IL-1β, TNF-α mRNA expression levels in hDPSCs, and the peaks expression levels of above inflammatory cytokines were reached at 3 h –12 h (Fig. 2).

### IL-6 protein expression

**Fig. 3:**
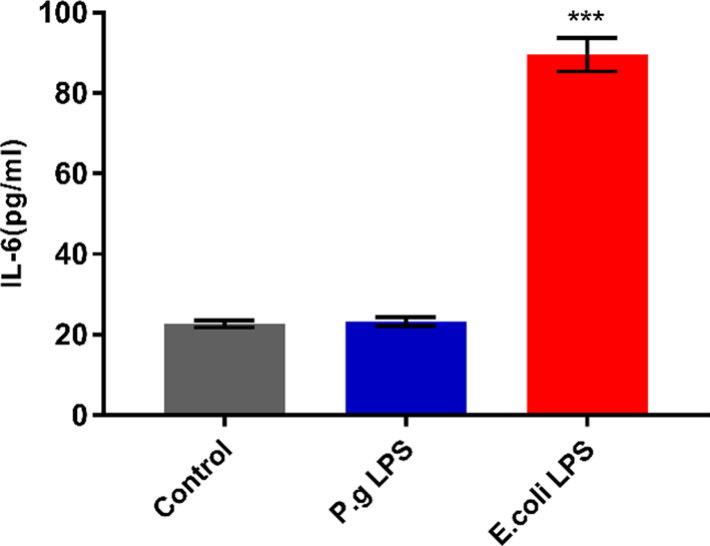
IL-6 protein concentration from the cell supernatant of hDPSCs through ELISA. The same concentration of LPS (1 μg/mL) from *E. coli*- or *P. gingivalis*-treated hDPSCs for 24 h. Data are shown as mean ± SD (n = 3). Cells without *E. coli* or *P. gingivalis* LPS treatment serve as the control group. ***p < 0.001

Results showed that the IL-6 protein production was significantly enhanced by *E. coli* LPS stimulation (*p* < 0.05). However, the protein production of IL-6 remained low in the 1 μg/mL *P. gingivalis* LPS stimulation group, and this finding was similar to that in the control group (*p* > 0.05, Fig. 3).

### TLR4 and TLR2 expression reactions to *E. coli *or *P. gingivalis* LPS

**Fig. 4:**
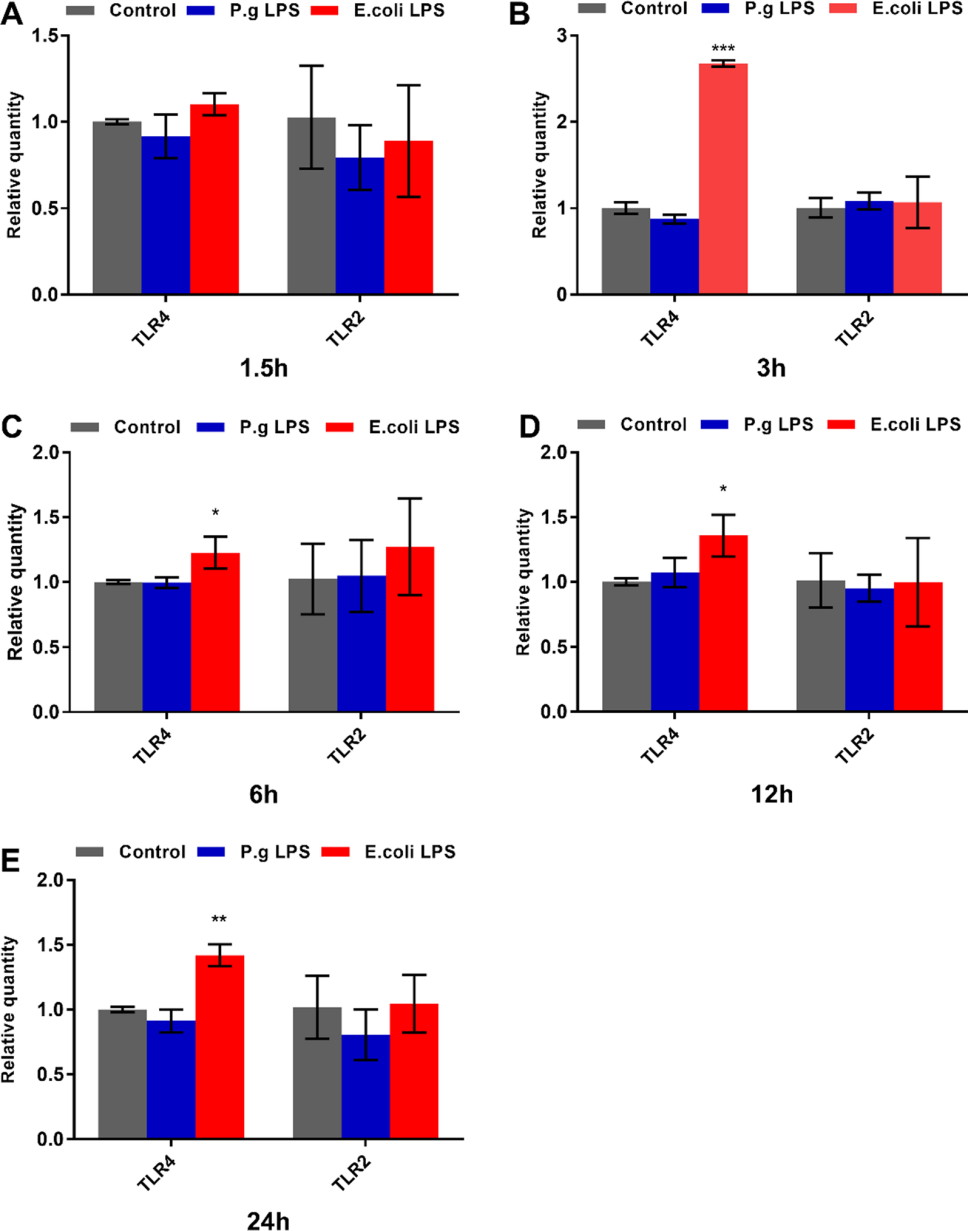
TLR4 and TLR2 mRNA expression in hDPSCs elicited by *E. coli* or *P. gingivalis* LPS. Cells are motivated by *E. coli* or *P. gingivalis* LPS (1 μg/mL). Cells without LPS stimulus serve as the control group. TLR4 and TLR2 on mRNA levels at **A** 1.5 h, **B** 3 h. **C** 6 h, **D** 12 h, and **E** 24 h. Data are shown as mean ± SD (n = 3). *p < 0.05, **p < 0.01, ***p < 0.001

**Fig. 5:**
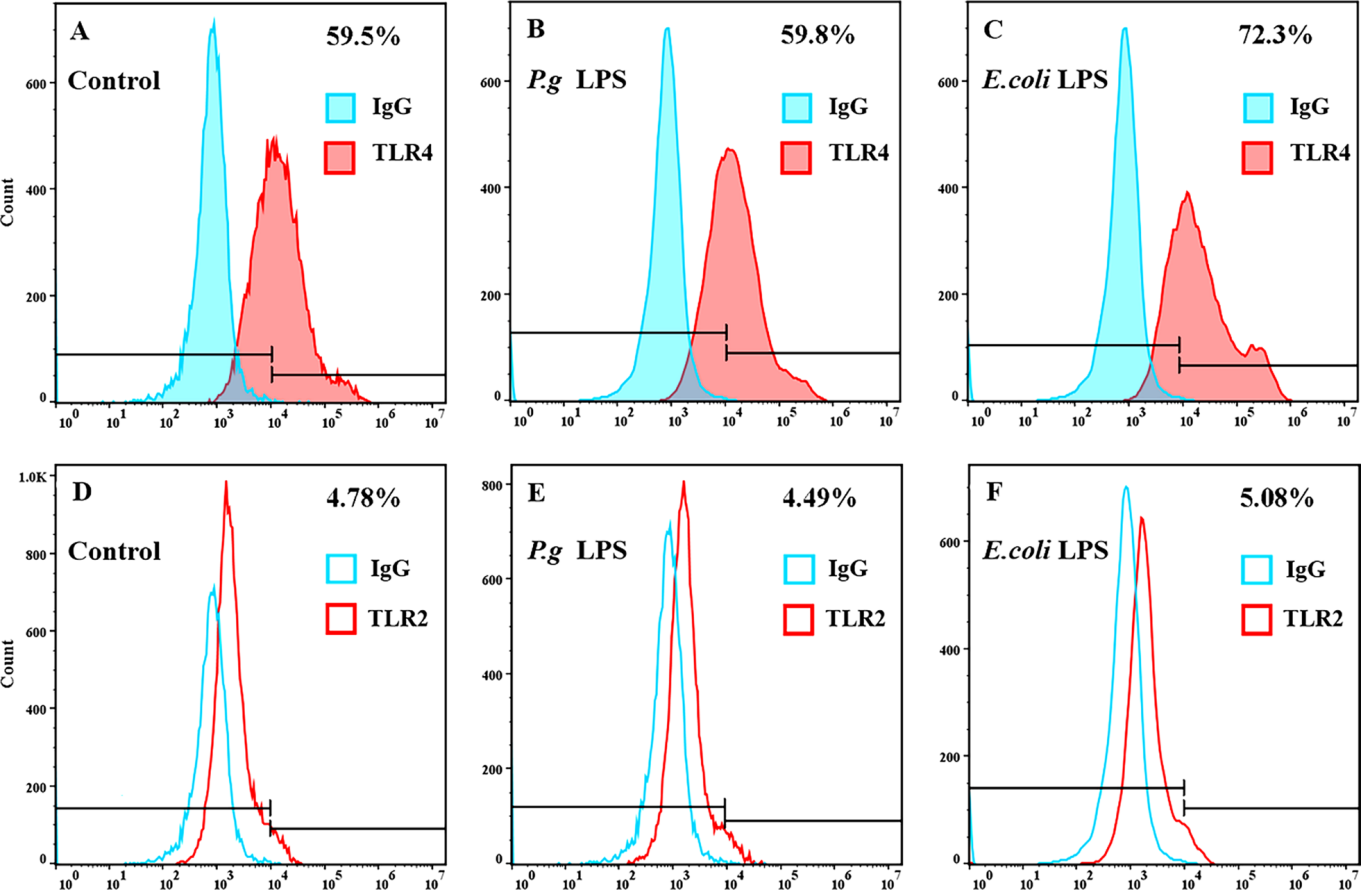
Production of TLR4 and TLR2 proteins on the surface of hDPSCs by flow cytometry. Cells are stimulated with *E. coli* or *P. gingivalis* LPS (1 μg/mL) for 24 h. Cells without LPS stimulus serve as the control group. Expression levels of **A**–**C** TLR4 and **D**–**F** TLR2 on the cell surface. Red-filled or unfilled histograms refer to TLR4 and TLR2, respectively, whereas blue-filled or unfilled histograms refer to the Isotype control IgG

The TLR4 and TLR2 mRNA expression levels by hDPSCs were measured using QRT-PCR. Results showed that 1 μg/ mL *E. coli* LPS significantly increased the TLR4 gene expression at 3 h (*p* < 0.001, Fig. 4B), 6 h (*p* < 0.05, Fig. 4C), 12 h (*p* < 0.05, Fig. 4D), and 24 h (*p* < 0.01, Fig. 4E), respectively. Moreover, the relative expression fold of TLR4 mRNA in the *E. coli* LPS group was highest at 3 h compared with that in the control group, corresponding to the peak expression period of proinflammatory cytokines. Nevertheless, no significant change in the mRNA expression was observed in hDPSCs activated by 1 μg/ mL *P. gingivalis* LPS (*p* > 0.05, Fig. 4). The expression of TLR2 mRNA was altered by neither *E. coli* nor *P. gingivalis* LPS in hDPSCs (*p* > 0.05, Fig. 4). Then, the flow cytometry analysis further verified the results of QRT-PCR (Fig. 5). The TLR4 production increased on the surface of hDPSCs initiated by 1 μg/ mL *E. coli* LPS (Fig. 5A, C). In contrast, the TLR4 protein amount in the 1 μg/ mL *P. gingivalis* LPS group was similar to that in the control group (Fig. 5A, B). However, the TLR2 protein was maintained at a deficient level on the surface of hDPSCs stimulated by 1 μg/ mL LPS from *E. coli* or *P. gingivalis* (Fig. 5D–F).

### TLR4 involved in the upregulation of inflammatory cytokines in hDPSCs by* E. coli* LPS

**Fig. 6:**
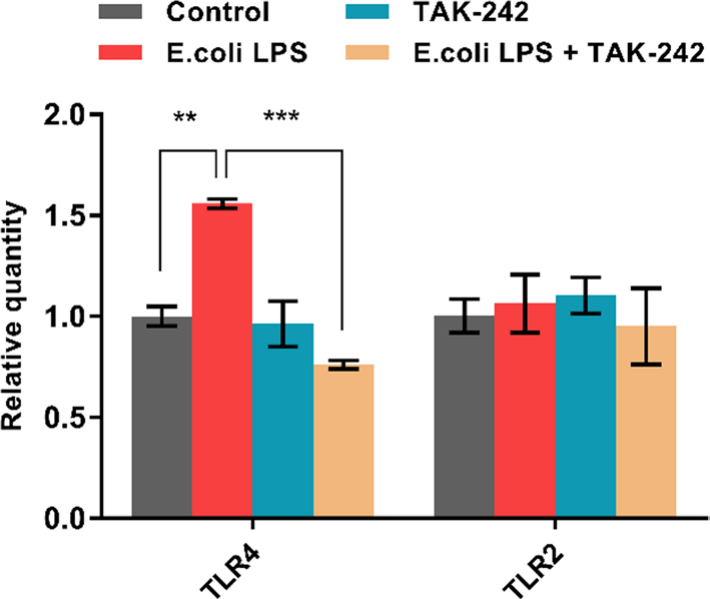
TAK-242 on *E. coli* LPS-induced TLR4 and TLR2 mRNA expression in hDPSCs. Treatment groups are added with TAK-242 in advance for 30 min and exposed to 1 μg/mL *E. coli* LPS for another 3 h. Cells without LPS stimulus and TAK-242 serve as the control group. Lines above the bar connect the two groups with statistical differences marked with star symbols. Data used are expressed as mean ± SD (n = 3). **p < 0.01, ***p < 0.001

**Fig. 7:**
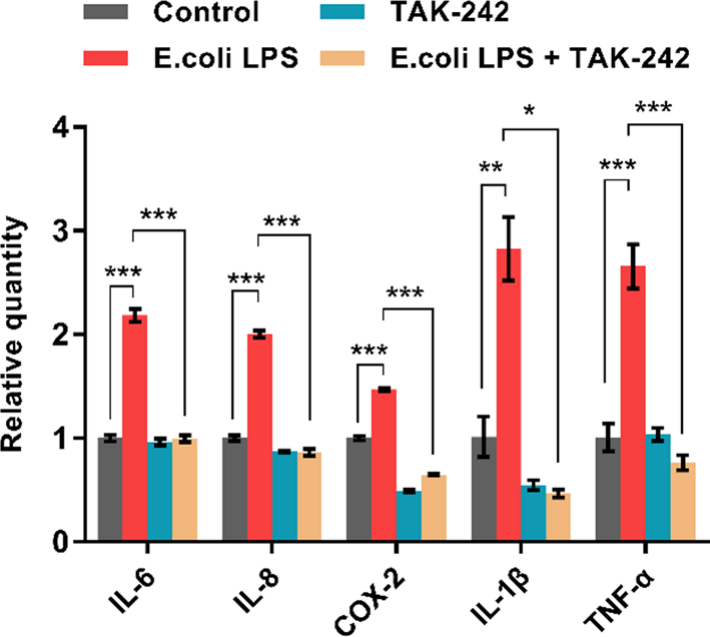
TAK-242 on the mRNA expression of *E. coli* LPS-induced inflammatory cytokines in hDPSCs. The hDPSCs are pretreated with or without TAK-242 for 30 min and exposed to 1 μg/mL *E. coli* LPS for another 3 h. Cells without LPS stimulus and TAK-242 serve as the control group. Lines above the bar connect the two groups with statistical differences marked with star symbols. Data used are expressed as mean ± SD (n = 3). *p < 0.05, **p < 0.01, ***p < 0.001

The TLR4 selective inhibitor TAK-242 was applied to confirm whether TLR4 participated in the expression of proinflammatory cytokines incited by 1 μg/ mL *E. coli* LPS in hDPSCs. First, our results revealed that 10 μmol/L TAK-242 could significantly block the expression of TLR4 in the group treated with *E. coli* LPS (*p* < 0.05) but did not influence the expression of TLR2 (*p* > 0.05, Fig. 6). As shown in Fig. 7, the pretreatment of TAK-242 significantly inhibited the *E. coli* LPS-induced expression of proinflammatory cytokines in hDPSCs, including IL-8, IL-6, COX-2, IL-1β, and TNF-α (*p* < 0.05).

## Discussion

Our research displays different inflammatory cytokine patterns in hDPSCs induced by *E. coli* LPS and *P. gingivalis* LPS. Only the LPS from *E. coli* significantly increases the expression of proinflammatory cytokines in hDPSCs within 24 h. Consistently, *E. coli* LPS increases the TLR4 expression in hDPSCs. Our results suggested that *E. coli* LPS but not *P. gingivalis* LPS should be used to stimulate hDPSCs in establishing an in vitro model of pulpitis.

The expression levels of inflammatory cytokines can reflect the pathological state in dental pulp tissue [44]. IL-1β is one of the essential mediators of acute dental pulp inflammation, and the increase of IL-1β level in dental pulp tissue aggravates the pulp inflammation [45]. IL-6 is a classic type of proinflammatory cytokine that mediates pulp inflammation [46]. IL-6 production in inflamed dental pulp tissues is significantly higher than in healthy tissues [47]. TNF-α, as an indicator of early pulp inflammation, plays a vital role in the pulp immune response [48]. IL-8 shows rapid chemotaxis and recruits immune cells to the inflammatory site [49], and COX-2 can induce vascular endothelial growth factor, thus promoting pulp inflammation [50].

The time-dependent expression pattern of cytokines in hDPSCs by LPSs is conducive to determining an optimal stimulation time to establish an in vitro model of pulpitis. Our research has compared the expression patterns of inflammatory mediators in hDPSCs induced by LPSs within 24 h. The peaks of all observed inflammatory mediators’ expression are unanimously reached 3–12 h after stimulation by 1 μg/mL E. coli LPS. These results are in line with those of previous studies [17, 42]. The types, concentrations, and stimulation times of stimuli used are different in earlier studies on potential pulp capping agents with anti-inflammatory effects. These differences are not suitable for comparing the anti-inflammatory effects of various potential pulp capping molecules. Our results are beneficial to establishing a baseline level of in vitro model of pulp inflammation to furtherly develop and screen new potential pulp capping agents with outstanding anti-inflammatory quality.

Compared with LPS from *P. gingivalis,* our results show that only the LPS from *E. coli* is a potent stimulator of proinflammatory cytokines in hDPSCs, which are in line with many previous studies [51–53]. An earlier study describes that the production of IL-6, IL-1β, and TNF-α from THP-1 cells and human monocytes stimulated by the *E. coli* LPS are relatively higher than those by the *P. gingivalis* LPS at 1, 10, 100, 1000, and 10,000 ng/ml [51]. Nebel et al. have compared the IL-6 expression by hPDLCs in response to LPS from *E. coli* or *P. gingivalis* and found that only the *E. coli* LPS is a competent stimulus [24]. Further studies reveal cell-specific response to LPSs of various bacterial origins. In another study, the LPS from *E. coli* induces strong chemokine and cytokine expression in the gingival fibroblasts, whereas the LPS from *P. gingivalis* elicits a strong reaction in macrophages [25].

Previous studies showed LPS with a concentration of 1 μg/ml is commonly used as a stimulus to induce inflammation [41, 42]. This concentration was found to be optimal in LPS inducing DPSCs inflammation in a previous study [43]. Our results show that *P. gingivalis* LPS (1 μg/mL) could not affect the tested inflammatory mediators’ expression in hDPSCs. Previous studies have reported different results exploring the inflammatory response of hDPSCs induced by *P. gingivalis* LPS [27, 31]. In the study by Ko YJ et al., 1, 5, 10, and 20 μg/mL *P. gingivalis* LPS significantly elevate the mRNA expression levels of IL-6 and TNF-α in a dose-dependent manner [38]. However, Ko YJ et al. have used *P. gingivalis* LPS in the laboratory extracted using the phenol/water method, which is different from the commercialized LPS in our study. Using the Northern blot analysis, Chang has demonstrated that *P. gingivalis* LPS rapidly induces IL-8 and IL-6 in dental pulp stem cells [27]. However, the *P. gingivalis* LPS in their research is donated by Dr. Arnold, who shows the *P. gingivalis* LPS is prepared in the laboratory by a hot phenol/water method [54]. Different preparations of LPS result in divergent contents of nucleic acid and protein impurities despite the similarity in structure [55]. Moreover, the expression of inflammatory mediators in the same cells induced by different preparations of LPS can be pretty differentiated [55]. Previous studies show that LPS structures have considerable heterogeneity among various bacterial species and activate host cells differently [33, 56]. The LPS from *P. gingivalis* is different in structure and function from *E. coli* [51]. The lipid A of *P. gingivalis* LPS lacks a phosphate group in the 4′ position and tetradecanoic acids but has long-chain fatty acids. Thus, the endotoxic activity of *P. gingivalis* LPS is relatively weak [53, 57]. Besides, *P. gingivalis* LPS is heterogeneous and has several lipid A species, including tri-, tetra-, and penta-acylated lipid As [58]. However, in their laboratory, all kinds of synthetic lipid As of LPS from *P. gingivalis* cannot induce intense inflammatory responses [57]. Moreover, tri- and tetra-acylated lipid As are even antagonistic in IL-8 and IL-6 expression [57]. Characteristic structures may be the part reason for the low potential of *P. gingivalis* LPS in inducing the inflammatory response of hDPSCs in our study.

However, Jung et al. show that the same commercialized *P. gingivalis* LPS from InvivoGen promotes IL-1β and IL-6 mRNA expression in human deciduous dental pulp cells [59], possibly related to the aging heterogeneity of hDPSCs in responses to *P. gingivalis* LPS. This finding is inconsistent with our results. Gingival fibroblasts show a considerable heterogeneity response to *P. gingivalis* LPS, which is reflected in increasing IL-6 expression on the mRNA level in gingival fibroblasts from some donors, remaining unchanged in gingival fibroblasts from the other donors [60]. The author speculates that heterogeneity can be due to the host cells’ different genetic backgrounds, ages, genders, and smoking status [60]. The *P. gingivalis* LPS seems not so stable as an inflammatory stimulus to fibroblasts. In our study, the hDPSCs separated from young permanent teeth rather than deciduous teeth are also a kind of fibroblast. We cannot eliminate the possibility of aging-individual heterogeneity of hDPSCs resulting in the poor bioactivity of *P. gingivalis* LPS in inducing the inflammatory response of hDPSCs in our study. Besides, the endotoxin activity of *P. gingivalis* LPS is susceptible to environmental factors, such as ATP, levels of hemin in the culture medium, Mg^2+^, ambient temperature, and pH [33, 61–64]. The above factors also may partly explain the inconsistency between the results of these studies.

Previous studies have documented TLRs, particularly TLR2 and TLR4 play a crucial role in regulating the intensity of the immune-inflammatory response during bacterial infection [65, 66]. Our data show that *E. coli* LPS increases the TLR4 expression level but not TLR2 in hDPSCs, whereas *P. gingivalis* LPS does not affect TLR4 or TLR2 expression. This result may suggest that the LPS from *P. gingivalis* may activate neither TLR2 nor TLR4 in hDPSCs and that TLR4 plays a pivotal role in the inflammatory reaction to *E. coli* LPS in hDPSCs.

TAK-242, a small-molecule derivative of cyclohexene, can selectively inhibit TLR4 signaling [67]. In a previous study, 10 μmol/L TAK-242 exclusively suppresses the TLR4-mediated cytokine production without inhibitory effect on other TLRs, such as TLR2, TLR3, or TLR9 in RAW264.7 cells [68]. In our study, TAK-242 at the same concentration also selectively blocks the activation of TLR4 in hDPSCs treated by *E. coli* LPS. The expression of all *E. coli* LPS-induced inflammatory mediators is dramatically suppressed by TAK-242. These data collectively imply that the LPS from *E. coli*, but not *P. gingivalis*, is a potent stimulus to propel the production of inflammatory cytokines by hDPSCs via the TLR4 signaling.

However, the current study’s limitations cannot precisely explain the inability of *P. gingivalis* LPS to elicit inflammatory reactions in hDPSCs in our research. Aside from the structural differences of *P. gingivalis* LPS caused by different synthetic methods, the interindividual heterogeneities of hDPSCs can be regarded as a possible reason. hDPSCs from volunteers should be collected and classified under differences in age, gender, lifestyle habits (such as smoking status), and genetic background. It would be interesting to determine the hDPSCs from volunteers with different conditions responding to *P. gingivalis* LPS individually and conclude whether individual differences cause it and reveal its underlying mechanism.

Besides, taking account of *P. gingivalis* being characteristic of immune escape, *P. gingivalis* is regarded as a poor inflammatory mediator stimulus [69, 70]. However, it has a solid capability to invade the tissue to avoid the phagocytosis of host immune cells and efficiently cause chronic inflammation [71]. *P. gingivalis* has been detected in root canal in irreversible pulpitis and periapical periodontitis [72]. *P. gingivalis* may enter the pulpal tissue through periapical foramen, lateral canal, or dentinal tubules [73]. Studies have shown that root scaling with hand instruments may facilitate bacterial penetration of *P. gingivalis* through dentinal tubules [74].

As LPS stimulation at early stages of pulp inflammation contributes to migration and differentiation of MSCs [3] leading to possible slowing or arresting or even reversal of pulpitis, the weak inflammatory-inducing compacity of *P. gingivalis* LPS observed in our study might explain the relatively advanced stages of endodontic lesion in which *P. gingivalis* has been detected. And, we can not rule out the possibility that the low induction of inflammatory mediators by *P. gingivalis* LPS in dental pulp may also be due to insufficient activation of the host immune response. So that to escape the monitoring of immune cells, it is easier to enter the pulp tissue for *P. gingivalis* LPS. Then it is more likely to cause chronic irreversible inflammation. The above hypothesis needs to be elucidated with more data in the future.

In conclusion, our research displays that *E. coli* LPS is a more stable and more potent stimulus than *P. gingivalis* LPS in producing inflammatory mediators in hDPSCs. The cytokine expression patterns induced by LPS in hDPSCs may help target treatment for inflammation mediators in pulpitis. Besides, our data suggest that TLR4 acts as an essential signaling intermediate between exogenous *E. coli* LPS and hDPSCs inflammatory reaction.

## Supplementary Information

**Additional file 1: Table S1**. The Primer sequences used for QRT-PCR.

**Additional file 2: Fig. S1**. Higher concentration of *P. gingivalis* LPS effect on proinflammatory cytokines in hDPSCs.

## Data Availability

The primer sequences generated during the current study are available in the supplementary material. The datasets generated and/or analysed during the current study are not publicly available due to data subject to third party restrictions but are available from the corresponding author on reasonable request.

## Abbreviations

COX-2: Cyclooxygenase-2
ELISA: Enzyme-linked Immunosorbent Assay
*E. coli*: *Escherichia coli*
hDPSCs: Human dental pulp stem cells
hPDLCs: Human periodontal ligament cells
h: Hour
GAPDH: Glyceraldehyde-3-phosphate dehydrogenase
IL-6: Interleukin-6
IL-1β: Interleukin-1β
IL-8: Interleukin-8
LPS: Lipopolysaccharide
LPSs: Lipopolysaccharides
MCP-1: Monocyte chemotactic protein-1
NF-κB: Nuclear factor kappa B
*P. gingivalis* (*P.g*): *Porphyromonas gingivalis*
QRT-PCR: Quantitative real-time polymerase chain reaction
TLRs: Toll-like receptors
TLR4: Toll-like receptor 4
TLR2: Toll-like receptor 2
TNF-α: Tumor necrosis factor-alpha
