# Supplementary material for: Different expression patterns of inflammatory cytokines induced by lipopolysaccharides from Escherichia coli or Porphyromonas gingivalis in human dental pulp stem cells
Source: BMC Oral Health. 2022 Apr 12;22:121. doi: 10.1186/s12903-022-02161-x (PMC9004173; doi:10.1186/s12903-022-02161-x)
Supplement: Supplementary file 1 — Additional file 1: Table S1. The Primer sequences used for QRT-PCR. [file 12903_2022_2161_MOESM1_ESM.docx]

**Table S1: Primer sequences used for RT-PCR**

| Genes | Sequences (5′–3′) |
| --- | --- |
| IL-1β | 5′-TGCACGATGCACCTGTACGA-3′  5′-AGGCCCAAGGCCACAGGTAT-3′ |
| TNF-α | 5′-CAGAGGGAAGAGTTCCCCAG-3′  5′-CCTCAGCTTGAGGGTTTGCTAC-3′ |
| IL-6 | 5′-GTGAGGAACAAGCCAGAGC-3′  5′-TACATTTGCCGAAGAGCC-3′ |
| IL-8 | 5′-TTTTGCCAAGGAGTGCTAAAGA-3′  5′-AACCCTCTGCACCCAGTTTTC-3′ |
| COX-2 | 5′-CTGGCGCTCAGCCATACAG-3′  5′-ACACTCATACATACACCTCGGT-3′ |
| TLR4 | 5′-CTGCAATGGATCAAGGACCA-3′  5′-TTATCTGAAGGTGTTGCACATTCC-3′ |
| TLR2 | 5′-GGGTTGAAGCACTGGACAAT-3′  5′-TCCTGTTGTTGGACAGGTCA -3′ |
| GAPDH | 5′-ATGGGGAAGGTGAAGGTCG-3′  5′-GGGGTCATTGATGGCAACAATA-3′ |
