# Supplementary material for: Different expression patterns of inflammatory cytokines induced by lipopolysaccharides from Escherichia coli or Porphyromonas gingivalis in human dental pulp stem cells
Source: BMC Oral Health. 2022 Apr 12;22:121. doi: 10.1186/s12903-022-02161-x (PMC9004173; doi:10.1186/s12903-022-02161-x)
Supplement: Supplementary file 2 — Additional file 2: Fig. S1. Higher concentration of P. gingivalis LPS effect on proinflammatory cytokines in hDPSCs. [file 12903_2022_2161_MOESM2_ESM.pdf]

Supplementary Materials

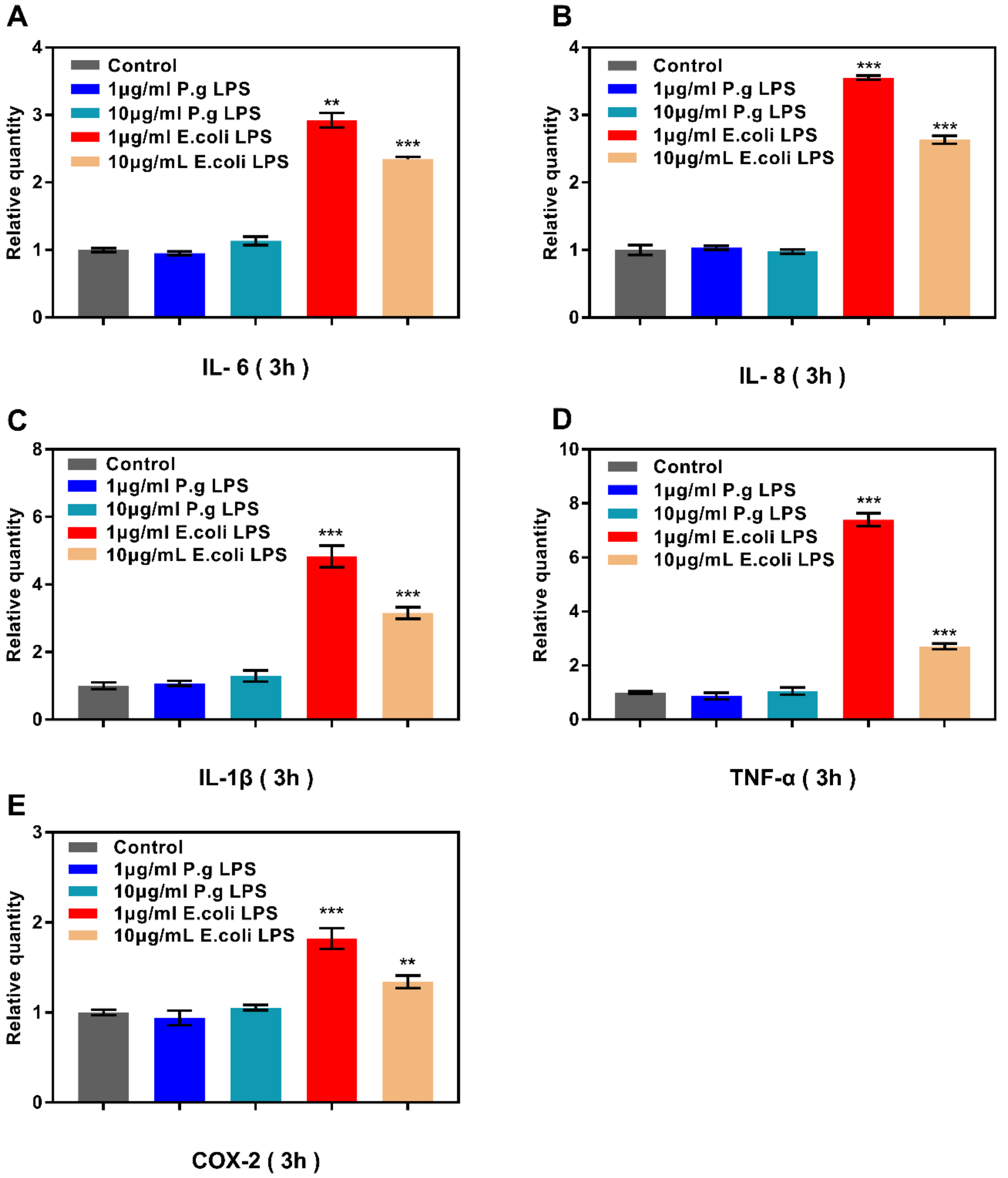

Figure S1. mRNA expression patterns of inflammatory cytokines in hDPSCs triggered by LPS (1 and 10 μg/ml) from *E. coli* or *P. gingivalis* determined using QRT-PCR. (A) IL-6, (B) IL-8, (C) COX-2 (D) IL-1β, and (E) TNF-α mRNA. Data are shown as mean ± SD ($n = 3$). **p < 0.01, ***p < 0.001.
